# Association of Food Deserts and Food Swamps With Obesity-Related Cancer Mortality in the US

**DOI:** 10.1001/jamaoncol.2023.0634

**Published:** 2023-05-04

**Authors:** Malcolm Seth Bevel, Meng-Han Tsai, April Parham, Sydney Elizabeth Andrzejak, Samantha Jones, Justin Xavier Moore

**Affiliations:** 1 Cancer Prevention, Control and Population Health, Georgia Cancer Center, Department of Medicine, Medical College of Georgia, Augusta University, Augusta; 2 Georgia Prevention Institute, Medical College of Georgia, Augusta University, Augusta; 3 Institute of Preventive and Public Health, Medical College of Georgia, Augusta University, Augusta

## Abstract

**Importance:**

Obesity-related cancers account for 40% of all cancers in the US. Healthy food consumption is a modifiable factor shown to reduce obesity-related cancer mortality, but residing in areas with less access to grocery stores (food deserts) or higher access to fast food (food swamps) reduces healthy food access and has been understudied.

**Objective:**

To analyze the association of food deserts and food swamps with obesity-related cancer mortality in the US.

**Design, Setting, and Participants:**

This cross-sectional ecologic study used US Department of Agriculture Food Environment Atlas data from 2012, 2014, 2015, 2017, and 2020 and Centers for Disease Control and Prevention mortality data from 2010 to 2020. A total of 3038 US counties or county equivalents with complete information on food environment scores and obesity-related cancer mortality data were included. An age-adjusted, generalized, mixed-effects regression model was performed for the association of food desert and food swamp scores with obesity-related cancer mortality rates. Data were analyzed from September 9, 2022, to September 30, 2022.

**Exposures:**

Food swamp score was calculated as the ratio of fast-food and convenience stores to grocery stores and farmers markets. Higher food swamp and food desert scores (20.0 to ≥58.0) indicated counties with fewer healthy food resources.

**Main Outcomes and Measures:**

Obesity-related cancer (based on the International Agency for Research on Cancer evidence between obesity and 13 types of cancer) mortality rates were categorized as high (≥71.8 per 100 000 population) vs low (<71.8 per 100 000 population) per county.

**Results:**

A total of 3038 counties or county equivalents with high obesity-related cancer mortality rates had a higher percentage of non-Hispanic Black residents (3.26% [IQR, 0.47%-26.35%] vs 1.77% [IQR, 0.43%-8.48%]), higher percentage of persons older than 65 years (15.71% [IQR, 13.73%-18.00%] vs 15.40% [IQR, 12.82%-18.09%]), higher poverty rates (19.00% [IQR, 14.20%-23.70%] vs 14.40% [IQR, 11.00%-18.50%]), higher adult obesity rates (33.00% [IQR, 32.00%-35.00%] vs 32.10% [IQR, 29.30%-33.20%]), and higher adult diabetes rates (12.50% [IQR, 11.00%-14.20%] vs 10.70% [IQR, 9.30%-12.40%]) compared with counties or county equivalents with low obesity-related cancer mortality. There was a 77% increased odds of having high obesity-related cancer mortality rates among US counties or county equivalents with high food swamp scores (adjusted odds ratio, 1.77; 95% CI, 1.43-2.19). A positive dose-response relationship among 3 levels of food desert and food swamp scores and obesity-related cancer mortality was also observed.

**Conclusions and Relevance:**

The findings of this cross-sectional ecologic study suggest that policy makers, funding agencies, and community stakeholders should implement sustainable approaches to combating obesity and cancer and establishing access to healthier food, such as creating more walkable neighborhoods and community gardens.

## Key Points

**Question:**

What are the odds of high obesity-related cancer mortality rates in US counties with low-income food desert or food swamp environments?

**Findings:**

In this ecologic cross-sectional study that included 3038 counties or county equivalents, those with the highest food swamp score had a 77% increased odds of high obesity-related cancer mortality.

**Meaning:**

Findings suggest that the increase in the creation and use of food swamps may be of national significance, warranting holistic methods of combating obesity and cancer through providing healthier food options.

## Teaser

This cross-sectional study investigates food deserts and food swamps in US counties and their association with obesity-related cancer mortality.

## Introduction

Cancer continues to be a formidable public health concern both in the US and internationally. In 2022 alone, it was projected that more than 1.9 million people in the US would receive a diagnosis of cancer, and among current survivors, an estimated 609 000 individuals will die from it.^1^ Major risk factors associated with carcinogenesis are either nonmodifiable (eg, age, sex, and genetics) or modifiable through healthy lifestyle changes (eg, reduced consumption of alcohol, tobacco abstinence, exercise, and dietary patterns).^2,3,4^ Of these modifiable risk factors, obesity and overweight status due to lack of exercise and healthy diet have been associated with 13 types of cancer according to the International Agency for Research on Cancer (endometrial, esophageal adenocarcinoma, gastric cardia, liver, kidney, multiple myeloma, meningioma, pancreatic, colorectal, gallbladder, breast, ovarian, and thyroid), which accounts for 40% of all cancers diagnosed in the US each year.^5^ Pathologic mechanisms associated with obesity continue to be explored but have been largely associated with increased chronic inflammation, hormonal disruption, and changes to the microbiome of the gut.^6^ The elevated chronic inflammatory environment due to obesity may lead to an increased risk of cancer mortality.^7,8,9^

Increased heathy eating has been associated with reduced risk of obesity and cancer incidence and mortality.^10,11,12,13,14,15^ One key barrier to healthy lifestyle access is residing in a food desert or food swamp.^16^ Food deserts are geographic regions where persons live more than 1.6 km (1 mile) from a supermarket and lack healthy food options. However, food swamps are unique in that these communities are more than 1.6 m (1 mile) from supermarkets and healthy food options and have more proinflammatory food options (eg, corner stores and fast-food restaurants) compared with fresh food options or no options.^17,18^ Although some studies have shown a larger promotion of unhealthy food options in African American communities compared with predominantly White communities,^19,20,21,22,23,24,25^ other studies have found that underserved populations are more willing to consume healthy foods when given equitable access to them.^26,27^

Food deserts and food swamps mainly exist in the South or Southeastern region, where chronic disease rates are the highest among US adults,^28,29^ including clusters for breast, lung, colorectal, and prostate cancers.^30,31^ Only 1 US study, to our knowledge, examined the association of food deserts with breast and colorectal cancers,^32^ finding that patients residing in food deserts had 16% increased 5-year breast cancer mortality risk and 12% increased 5-year colorectal cancer mortality risk compared with patients not residing in food deserts.^32^ A different US study found that food swamps were strongly correlated with obesity compared with food deserts alone.^17^ Finally, another US study found that patients with esophageal cancer who resided in food deserts had a more than 6-fold increased risk of hospital readmission after esophagectomy.^33^ There is a paucity of research on the association of food deserts and food swamps with obesity-related cancer morbidity and mortality, which calls for additional research to comprehensively characterize this association. The purpose of this study was to examine the association of food deserts and food swamps with obesity-related cancer mortality among all US counties in the past decade.

## Methods

### Ethical Review of the Study

This analysis was considered exempt by Augusta University from undergoing institutional review board review and receiving informed consent, given that the data were deidentified and publicly available. All analyses were performed according to relevant regulations and the Strengthening the Reporting of Observational Studies in Epidemiology (STROBE) reporting guideline.

### Study Design

We conducted a cross-sectional study at the ecologic level among US counties (n = 3142), using 2010 to 2020 county-level cancer mortality data from the Centers for Disease Control and Prevention (CDC)^34^ and 2012, 2014, 2015, 2017, and 2020 county-level food environment data from the Economic Research Service of the US Department of Agriculture.^35^ We elected these respective time periods to provide robust estimation of county-level food environment measures at multiple levels. The county-level CDC cancer mortality data include multiple causes of cancer death (esophagus adenocarcinoma, gastric cardia, colon and rectum, liver, gallbladder, pancreas, postmenopausal breast, corpus uteri, ovary, kidney, meningioma, thyroid, and multiple myeloma) for noninstitutionalized adults aged 18 years or older and residing in the US.^34^ We linked the county-level mortality data to the county-level food environment data via the Food Environment Atlas (FEA) by the available Federal Information Processing System Codes.

### Study Sample

**Figure 1.**
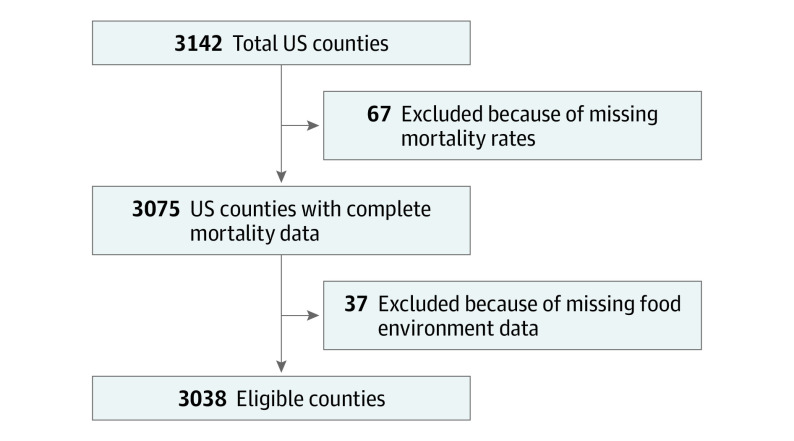
Flowchart of US Counties or County Equivalents Reporting Food Environment Measures

The FEA and CDC mortality data had 3142 US counties or county equivalents^36^ (henceforth counties), respectively. To obtain an eligible sample for our study, we excluded counties with missing data for mortality rates (n = 67) and missing food environment factors, including incomplete data on fast-food restaurants or grocery stores (n = 37). The final sample size for this analysis included 3038 US counties with complete FEA and CDC mortality data (Figure 1).

### Obesity-Related Cancer Mortality: Outcome of Interest

The outcome of interest (obesity-related cancer mortality) between 2010 and 2020 was based on *International Statistical Classification of Diseases and Related Health Problems, Tenth Revision* codes for underlying cause of death (eTable 1 in Supplement 1) and findings from the International Agency for Research on Cancer Handbook Working Group.^5^ Death certificates, which include a record for every death of a US resident, provided the underlying data for cause of death. Overweight status was defined as having a body mass index (calculated as weight in kilograms divided by height in meters squared) of 25 to 29.9, and obesity status was defined as having a body mass index of 30 or higher.^37^ Given that overweight status, obesity status, or both are a major risk factor for multiple cancer types, we used the age-adjusted mortality rates (per 100 000 population) from the CDC that consisted of all 13 obesity-related cancer types. Furthermore, we categorized the continuous cancer mortality rates as high (≥71.8 per 100 000 population) vs low (<71.8 per 100 000 population) according to the median of county-level mortality rates.

### Food Deserts and Food Swamps: Primary Exposures of Interest

Food desert and food swamp scores (also considered food environment measures) were the key exposures of interest. County-level data (2012, 2014, 2015, 2017, and 2020) from the FEA were used to examine the association between food choices and causes of cancer death in the US. The FEA data include updated county-level food environment factors (eg, store count based on the North American Industry Classification System, store or restaurant proximity, and food and nutrition assistance programs) and demographic and clinical characteristics, such as county obesity and diabetes prevalence rates.^35^ Food deserts were calculated through the FEA as the proportion of each county’s total population identified as having both low income and low access to grocery stores. Low income was defined as having a household income less than or equal to 200% of the federal poverty threshold, and low access to grocery stores was defined as living more than 1.6 km (1 mile) from a supermarket or grocery store in an urban area or more than 16 km (10 miles) in a rural area.^38,39^ The food swamp score was calculated as the ratio of fast-food restaurants and convenience stores (eg, corner stores) to grocery stores and farmers markets. This score was modified from the traditional Retail Food Environment Index score.^17^ Each county had its respective scores calculated for 2012, 2014, 2015, 2017, and 2020; then the scores were aggregated over the 10-year period of the cancer mortality data to estimate continuous food swamp and food desert scores from 2010 to 2020. After the continuous measures were calculated, we categorized food desert and food swamp scores into low, moderate, and high scores based on the distribution (tertiles) of the total scores per county. Higher food swamp and food desert scores (20.0 to ≥58.0) indicate counties with fewer healthy food resources.

### Covariates

Other covariates of interest reported and considered for analysis were obtained from the FEA according to their latest year of availability. The covariates included race and ethnicity (percentage of each racial group per county in 2010: non-Hispanic American Indian or Alaska Native, non-Hispanic Asian, non-Hispanic Black, non-Hispanic Hawaiian or Pacific Islander, Hispanic, and non-Hispanic White), age (percentage of the population ≥65 years in 2010), median household income in 2015, poverty rate in 2015 (per 100 000 population), adult obesity rates per county in 2017 (per 100 000 population), and adult diabetes rates per county in 2013 (per 100 000 population).

### Statistical Analysis

Analysis of variance was used to assess differences in demographic and clinical characteristics among counties with high or low obesity-related cancer mortality rates. Pearson correlation coefficients were used to evaluate the association between the measured food environments and cancer mortality rates. We performed a generalized linear mixed model accounting for an unstructured covariance-variance matrix to examine the association between county-level food swamps (and deserts) and obesity-related cancer mortality to account for possible between-county correlation using a binomial distribution and logit function. The exponentiated effects from this model may be interpreted as the odds ratios and associated 95% CIs of the association between the food environment measures and obesity-related cancer mortality. Additionally, a multilevel generalized linear mixed-effects model was used to analyze the association among 3 levels of food desert scores, 3 levels of food swamp scores, and 3 levels of obesity-related cancer mortality rates (low, moderate, and high). Results from this polytomous model can be interpreted as the log odds of counties with either high or moderate obesity-related cancer mortality rates compared with the log odds of counties with low mortality rates (referent category). All models were reported as adjusted odds ratios (AORs) and associated 95% CIs, with statistical significance set at .05 and the *P* values based on 2-sided *t* tests. All analyses were conducted with SAS, version 9.4 (SAS Institute). Data were analyzed from September 9, 2022, to September 30, 2022.

## Results

**Figure 2.**
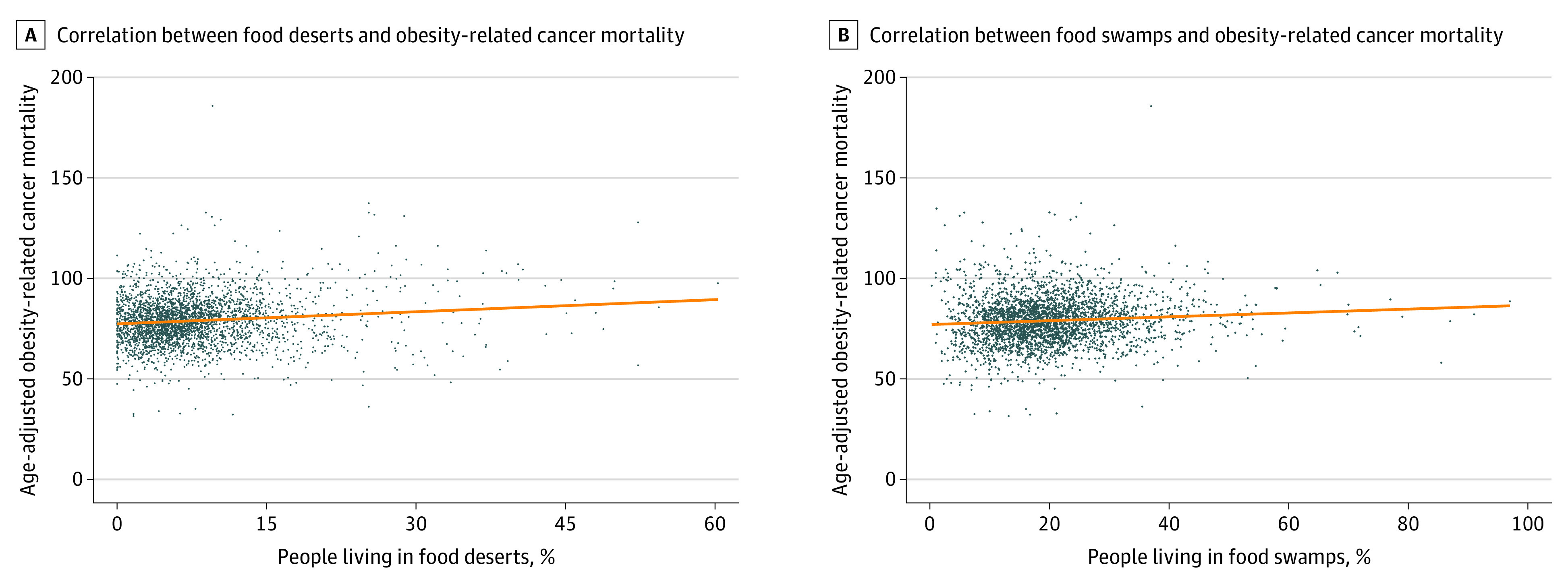
Correlations Between Food Deserts and Obesity-Related Cancer Mortality and Between Food Swamps and Obesity-Related Cancer Mortality A, Correlation between total percentage of people living in food deserts and age-adjusted obesity-related cancer mortality (ρ = 0.12). B, Correlation between food swamp scores and age-adjusted obesity-related cancer mortality (ρ = 0.08).

Of the 3142 counties in the US, a total of 3038 (96.7%) were included in this analysis, of which 758 (25.0%) had high (within the fourth [highest] quartile) obesity-related cancer mortality. These specific counties had a higher percentage of non-Hispanic Black residents (3.26% [IQR, 0.47%-26.35%] vs 1.77% [IQR, 0.43%-8.48%]), higher percentage of persons older than 65 years (15.71% [IQR, 13.73%-18.00%] vs 15.40% [IQR, 12.82%-18.09%]), higher poverty rates (19.00% [IQR, 14.20%-23.70%] vs 14.40% [IQR, 11.00%-18.50%]), higher adult obesity rates (33.00% [IQR, 32.00%-35.00%] vs 32.10% [IQR, 29.30%-33.20%]), and higher adult diabetes rates (12.50% [IQR, 11.00%-14.20%] vs 10.70% [IQR, 9.30%-12.40%]) compared with counties with low obesity-related cancer mortality (Table 1). Furthermore, these counties had higher percentages of persons residing in food deserts (7.39% [IQR, 4.09%-11.65%] vs 5.99% [IQR, 3.47%-9.50%]) and food swamps (19.86% [IQR, 13.91%-26.40%] vs 18.20% [IQR, 13.14%-24.00%]) compared with counties with low cancer mortality. The correlation analysis (Figure 2) observed that both food desert and food swamp scores were positively correlated with obesity-related cancer mortality, with the correlation being slightly higher between food deserts and obesity-related cancer mortality (food deserts, ρ = 0.12; food swamps, ρ = 0.08). Nevertheless, food desert and food swamp index scores were determined to be unique, given the low coefficients.

**Table 1.** Descriptive and Clinical Characteristics Among 3038 US Counties or County Equivalents

Study characteristics	Age-adjusted obesity-related cancer mortality rates, median (IQR)		*P* value^c^
	Low (n = 2289)^a^	High (n = 758)^b^	
Demographic characteristics			
Race and ethnicity, %			
Non-Hispanic American Indian or Alaska Native	0.31 (0.20-0.63)	0.27 (0.18-0.51)	<.001
Non-Hispanic Asian	0.52 (0.30-1.19)	0.35 (0.23-0.57)	<.001
Non-Hispanic Black	1.77 (0.43-8.48)	3.26 (0.47-26.35)	<.001
Non-Hispanic Hawaiian or Pacific Islander	0.03 (0.01-0.05)	0.02 (0.01-0.03)	.03
Hispanic	3.69 (1.77-9.35)	2.34 (1.30-5.21)	<.001
Non-Hispanic White	86.11 (70.30-94.07)	82.85 (58.71-94.29)	<.001
Population aged ≥65 y, %	15.40 (12.82-18.09)	15.71 (13.73-18.00)	.02
Income			
Household income, No. (SD), $	48 598 (12 692)	41 783 (8915)	<.001
Poverty rate, %	14.40 (11.00-18.50)	19.00 (14.20-23.70)	<.001
Clinical characteristics, %			
Adult obesity rate	32.10 (29.30-33.20)	33.00 (32.00-35.00)	<.001
Adult diabetes rate	10.70 (9.30-12.40)	12.50 (11.00-14.20)	<.001
Food environment, %			
Fast-food restaurants^d^	18.20 (6.20-60.00)	11.20 (4.40-25.00)	<.001
Convenience stores^d^	17.40 (8.00-42.60)	13.30 (6.80-25.20)	<.001
Grocery stores^d^	6.00 (3.00-14.80)	4.40 (2.40-8.00)	<.001
Supercenters^d^	1.00 (0.00-2.00)	0.60 (0.00-1.00)	<.001
Specialized food stores^d^	1.60 (0.20-5.40)	0.60 (0.00-2.00)	<.001
Farmers market^d^	1.20 (0.40-3.00)	0.80 (0.00-1.40)	<.001
Food desert^e^	5.99 (3.47-9.50)	7.39 (4.09-11.65)	<.001
Food swamp (comprehensive RFEI)	18.20 (13.14-24.00)	19.86 (13.91-26.40)	<.001

Abbreviation: RFEI, Retail Food Environment Index.

^a^ Low categorized as counties with obesity-related cancer mortality rates from 31.0 to 71.7 per 100 000 population.

^b^ High categorized as counties with obesity-related cancer mortality rates from 71.8 to 185.7 per 100 000 population.

^c^ Determined from 1-way analysis of variance.

^d^ Denotes the count of respective variable within each county.

^e^ Denotes the counties with percentage of residents with low income and low access to grocery stores.

The age-adjusted odds of counties having high obesity-related cancer mortality were elevated (77%) among counties with high food desert scores (AOR, 1.59; 95% CI, 1.29-1.94) and high food swamp scores (AOR, 1.77; 95% CI, 1.43-2.19) compared with low food environment measures (Table 2). Additionally, we observed a positive dose-response relationship (Table 3) between tertiles of the food environment measures and obesity-related cancer mortality, including greater than 2-fold increased odds of high obesity-related cancer mortality among counties with high food swamp scores (AOR, 2.10; 95% CI, 1.67-2.63) compared with counties with low food swamp scores. After adjusting for age, race, and poverty rate (eTable 2 in Supplement 1), we observed an almost 30% increased odds of high obesity-related cancer mortality among counties with high food swamp scores (AOR, 1.29; 95% CI, 1.03-1.63). However, additional adjustment by adult obesity rates showed no significance. Also, we observed nonsignificant associations in fully adjusted models between tertiles of the food environment measures and obesity-related cancer mortality (eTable 3 in Supplement 1).

**Table 2.** Association of Food Environment Measures With Obesity-Related Cancer Mortality Among 3038 US Counties or County Equivalents

Variable	Odds of high obesity-related cancer mortality		
	Counties with low obesity-related cancer mortality, No. (%) (n = 2283)^a^^,^^b^	Counties with high obesity-related cancer mortality, No. (%) (n = 758)^a^^,^^b^	AOR (95% CI)
Food desert			
Low	800 (35.0)	215 (28.4)	1 [Reference]
Moderate	781 (34.2)	235 (31.0)	1.12 (0.91-1.38)
High	708 (31.0)	307 (40.5)	1.59 (1.29-1.94)
Food swamp (comprehensive RFEI)			
Low	794 (34.8)	219 (28.9)	1 [Reference]
Moderate	785 (34.4)	229 (30.2)	1.15 (0.93-1.43)
High	708 (31.0)	306 (40.4)	1.77 (1.43-2.19)

Abbreviations: AOR, adjusted odds ratio; RFEI, Retail Food Environment Index.

^a^ Adjusted for the percentage of county population aged 65 years or older. Results from the adjusted generalized mixed-effects models can be interpreted as the odds of counties with high obesity-related cancer mortality rates compared with that of those with low mortality rates (referent category).

^b^ Group percentage presented as the proportion of counties within variable strata with food environment category.

**Table 3.** Multivariable Polytomous Association of Food Environment Measures With Obesity-Related Cancer Mortality Among 3038 US Counties or County Equivalents^a^

Variable	Low, %^b^^,^^c^	Moderate^d^		High^e^	
		%^c^	AOR (95% CI)	%^c^	AOR (95% CI)
Food desert					
Low	38.4	32.7	1 [Reference]	28.9	1 [Reference]
Moderate	29.7	38.4	1.52 (1.23-1.87)	31.9	1.43 (1.15-1.78)
High	31.8	29.4	1.06 (0.86-1.32)	38.8	1.59 (1.28-1.96)
Food swamp (comprehensive RFEI)					
Low	38.1	32.3	1 [Reference]	29.6	1 [Reference]
Moderate	33.8	35.7	1.33 (1.07-1.64)	30.5	1.29 (1.03-1.61)
High	28.1	32.5	1.50 (1.20-1.88)	39.4	2.10 (1.67-2.63)

Abbreviations: AOR, adjusted odds ratio; RFEI, Retail Food Environment Index.

^a^ Adjusted for the percentage of county population aged 65 years or older. Results from this polytomous generalized mixed-effects models can be interpreted as the log odds of counties with either high or moderate obesity-related cancer mortality rates compared with the log odds of counties with low mortality rates (reference category).

^b^ Low categorized as counties with obesity-related cancer mortality rates from 31.0 to 74.0 per 100 000 population.

^c^ Group percentage presented as the proportion of counties within variable strata with food environment category.

^d^ Moderate categorized as counties with obesity-related cancer mortality rates from 75.0 to 82.0 per 100 000 population.

^e^ High categorized as counties with obesity-related cancer mortality rates from 83.0 to 185.7 per 100 000 population.

## Discussion

Medical advancements have been associated with reduced cancer mortality rates in the US. However, prior studies showed an association of socioeconomic factors and social determinants of health with worse cancer outcomes.^32,40,41,42,43^ Among most US counties, we observed a 77% elevated odds of obesity-related cancer death among counties with higher food swamp scores. This was a higher magnitude of association vs the association between food deserts and obesity-related cancer mortality. Additionally, counties with the highest food swamp scores had a more than 2-fold increased risk of cancer mortality compared with counties with moderate or the lowest scores. Our results indicate that food swamps may be a more comprehensive and novel indicator of the typical US food environment compared with food deserts because the counties with the fewest healthy food resources had the highest odds of obesity-related cancer mortality. This is the first study, to our knowledge, to assess whether food desert and food swamp areas across the nation were associated with obesity-related cancer mortality among multiple cancer types in the past 10 years.

To date, only 3 studies have observed that different food environments are associated with obesity or obesity-related cancers. Specifically, these studies showed that food swamps are correlated with obesity alone,^17^ that persons residing in food deserts in California had higher mortality risk of 2 obesity-related cancer types (breast and colorectal),^32^ and that patients with esophageal cancer who resided in food deserts in New Hampshire had a higher risk of 30-day readmission after esophagectomy.^33^ Our findings mirror results from the only previous study analyzing food deserts and cancer mortality. Fong and colleagues^32^ found that patients with breast cancer who resided in food deserts had a 16% mortality risk and that patients with colorectal cancer who resided in food deserts had a 12% greater mortality risk. The observed elevated association in our study could be due to the difference in data sources (county level vs patient level) despite the fact that both respective studies used multiple years of cancer mortality data. Also, the observed association in our study could be due to an increase in obesity-related cancer deaths, likely explained by treatment complications (eg, surgery or chemotherapy) among patients with obesity vs patients without obesity.^44^

Stark increases in obesity rates occurred in the US between the late 1990s and 2017,^45^ threatening population health at epidemic proportions.^46^ Contemporaneous obesity research and growing policy-making concerns continue to focus on preventive interventions aimed at exploring and bringing awareness to the mechanisms of known associated factors, such as physical inactivity, improper diet or malnutrition, and specific environmental characteristics. As a result, researchers have begun to assess the role of the neighborhood environment and its potential association with obesity and overall health,^47^ including the consideration of food accessibility, acquisition, and consumption, and to what degree healthy food options are promoted.

The established existence of food deserts and the emergence of food swamps can be explained by a few theories. One proposed theory is that chain grocery stores (eg, Kroger, Sprouts, and Publix) lack a vested interest in remaining in urban neighborhoods with lower socioeconomic status (where most of the population is often composed of racial and ethnic minority groups) for longer periods, thus creating a food desert.^43^ Coupled with the increasing growth rate of fast-food restaurants in recent years^48,49^ and the intentional advertisement of unhealthy foods in urban neighborhoods with lower socioeconomic status,^19,20,23,24,50^ the food desert may transform into a food swamp. Another theory proposes competition issues between chain grocery stores and smaller grocery stores (ie, mom-and-pop shops). Finally, disparities regarding food-barren environments (including the excess promotion of unhealthy foods in communities with racial and ethnic minority groups) can be traced to the historic and resurging discriminatory practices of gentrification and redlining.^51^

Redlining is commonly known as the denial of services (eg, insurance, banking, supermarkets) to residents with lower socioeconomic status or to racial and ethnic minority groups either by not building resources in underserved neighborhoods or through the selective price gouging of goods.^51,52,53,54^ The results of these racially charged practices arguably resonate throughout the country and affect successive generations of racial and ethnic minority groups, particularly in the Southeast. Therefore, one could infer that disparities in access to healthy food options through the disenfranchisement of communities with lower socioeconomic status or of racial and ethnic minority groups are a probable augmenting factor regarding obesity and obesity-related cancer types. Future prospective studies should expound on the racial differences between residing in a food desert or food swamp and obesity-related cancer outcomes.

A distinct resource that can improve healthy food access in the US is community gardens.^55^ Although gardens span the country, garden building and maintenance surged during the 1970s and 1980s in the New England and West Coast regions of the US despite the strong agrarian culture in the South and Southeast and a longer growing season. Community gardening has been an essential aspect for healthy lifestyle movements, with various benefits such as increased physical activity, food intake, and communal engagement.^56^ Also, studies incorporating individual or community garden–based components for healthy lifestyle changes have primarily been conducted with children.^57,58,59^ Future clinical trials should implement an innovative healthy lifestyle intervention using neighborhood- and patient-level data along with the maintenance and use of community gardens among adults residing in food deserts or food swamps to better understand the growing concern of unhealthy food environments in the US.

### Strengths and Limitations

Findings from our analysis were the first of their kind, to our knowledge, and comprehensively delineated how the increase in of food swamps adversely affects obesity and obesity-related cancer outcomes. We had complete data on 3038 of the 3142 US counties (96.7%) and used a comprehensive scoring system to classify the difference between food deserts and food swamps. Our study has limitations, however. The ecologic design of the analysis did not account for temporal sequence; thus, no causal association of food deserts and food swamps with obesity-related cancer mortality exists. Results are based on groups instead of individuals, so interpretation should be made at the county level to avoid individual inferences of aggregate data (ie, committing the ecologic fallacy). The food environment measures were based on county reporting of the healthy and unhealthy food options available; misclassification of various food stores (and subsequent validity concerns) could have occurred because they were categorized by North American Industry Classification System codes.^60^ Last, information on race, ethnicity, and income was based on county-level data, which may limit the ability to further examine racial, ethnic, or socioeconomic differences at the individual level in the association of food deserts and food swamps with obesity-related cancer mortality.

## Conclusions

This cross-sectional study found that counties with food desert or food swamp environments had significantly greater odds of obesity-related cancer mortality. Food swamps appear to be a growing epidemic across the US, likely because of systemic issues, and should draw concern and conversation from local and state officials. Future studies should analyze the potential mediating association of obesity and specific social determinants of health with unhealthy food environments and cancer outcomes via group-level (Census tract or neighborhood-level areas) and individual-level data to provide a thorough illustration regarding specific social determinants of health and cancer. Also, researchers should consider clinical and mixed-methods studies to determine whether racial, sex, or socioeconomic status differences exist in the association between food desert or food swamp environments and cancer. Community-based participatory research efforts can include partnerships with local policy makers, community stakeholders (eg, farmers and gardeners), and funding agencies to create and maintain sustainable approaches to combating obesity and establishing healthier accessible foods, including community garden development in underserved communities nationwide.

## Notes

Supplement 1. **eTable 1.** *ICD-10* Codes for Identification of Obesity-Related Cancers **eTable 2.** Additional Generalized Mixed Effects Models for the Association of Food Environment Measures and Obesity-Related Cancer Mortality Among U.S. Counties (N = 3038) **eTable 3.** Fully Adjusted Multilevel Generalized Mixed Effects Models for the Association of Food Environment Measures and Obesity-Related Cancer Mortality among U.S. Counties (N = 3038)
eReferences.

Supplement 2.
Data Sharing Statement
