# Supplementary material for: Association of Food Deserts and Food Swamps With Obesity-Related Cancer Mortality in the US
Source: JAMA Oncol. 2023 May 4;9(7):909–16. doi: 10.1001/jamaoncol.2023.0634 (PMC10160992; doi:10.1001/jamaoncol.2023.0634)
Supplement: Supplement 1. — eTable 1. ICD-10 Codes for Identification of Obesity-Related Cancers eTable 2. Additional Generalized Mixed Effects Models for the Association of Food Environment Measures and Obesity-Related Cancer Mortality Among U.S. Counties (N = 3038) eTable 3. Fully Adjusted Multilevel Generalized Mixed Effects Models for the Association of Food Environment Measures and Obesity-Related Cancer Mortality among U.S. Counties (N = 3038) eReferences. [file jamaoncol-e230634-s001.pdf]

Supplementary Online Content

Bevel MS, Tsai MH, Parham A, Andrzejak SE, Jones S, Moore JX. Association of food deserts and food swamps with obesity-related cancer mortality in the US. *JAMA Oncol*. Published online May 4, 2023. doi:10.1001/jamaoncol.2023.0634

eTable 1. *ICD-10* Codes for Identification of Obesity-Related Cancers

eTable 2. Additional Generalized Mixed Effects Models for the Association of Food Environment Measures and Obesity-Related Cancer Mortality Among U.S. Counties (N = 3038)

eTable 3. Fully Adjusted Multilevel Generalized Mixed Effects Models for the Association of Food Environment Measures and Obesity-Related Cancer Mortality among U.S. Counties (N = 3038)

eReferences.

This supplementary material has been provided by the authors to give readers additional information about their work.

© 2023 Bevel MS et al. *JAMA Oncology*.

eTable 1. ICD-10 codes for identification of obesity-related cancers

ICD-10 Code	Definition and/or Technical Information
C15.9	Esophagus, unspecified – Malignant neoplasms
C16.0	Cardia – Malignant neoplasms
C18.9	Colon, unspecified – Malignant neoplasms
C22.0	Liver cell carcinoma – Malignant neoplasms
C23.0	Malignant neoplasm of gallbladder
C25.9	Pancreas, unspecified – Malignant neoplasms
C32.9	Larynx, unspecified – Malignant neoplasms
C50.9	Breast, unspecified – Malignant neoplasms
C54.9	Corpus uteri, unspecified – Malignant neoplasms
C56.0	Malignant neoplasm of ovary
C64.0	Malignant neoplasm of kidney, except renal pelvis
C73.0	Malignant neoplasm of thyroid gland
C90.0	Multiple myeloma – Malignant neoplasms

© 2023 Bevel MS et al. *JAMA Oncology.*

eTable 2. Additional Generalized Mixed Effects Models for the Association of Food Environment Measures and Obesity-Related Cancer Mortality Among U.S. Counties (N = 3038)

	Adjusted OR (and 95% CI) of High Obesity-Related Cancer Mortality			
**Variable**	Model 1	Model 2	Model 3	Model 4
**Food Desert**				
**Low**	1.00 (Referent)	1.00 (Referent)	1.00 (Referent)	1.00 (Referent)
**Moderate**	1.11 (0.89 – 1.38)	1.08 (0.86 – 1.35)	1.08 (0.86 – 1.35)	1.04 (0.83 – 1.31)
**High**	1.14 (0.92 – 1.41)	1.07 (0.86 – 1.34)	1.18 (0.95 – 1.47)	1.10 (0.87 – 1.38)
**Food Swamp (Comprehensive RFEI)**				
**Low**	1.00 (Referent)	1.00 (Referent)	1.00 (Referent)	1.00 (Referent)
**Moderate**	1.04 (0.83 – 1.31)	1.02 (0.81 – 1.29)	0.95 (0.76 – 1.20)	0.92 (0.72 – 1.17)
**High**	**1.29 (1.03 – 1.63)**	**1.32 (1.04 – 1.67)**	1.06 (0.84 – 1.34)	1.06 (0.83 – 1.36)

Model 1: Adjusted for the percentage of county population ages 65 years old or older, percentage of NH-Black residents per county, and poverty rate per county. Model 2: Adjusted for the percentage of county population ages 65 years old or older, percentage of NH-Black residents per county, poverty rate per county, and physician density. Model 3: additionally adjusted for adult obesity rate per county. Model 4: full model.

Results from the adjusted generalized mixed effects models can be interpreted as the odds of counties with high obesity-related cancer mortality rates compared to the odds of counties with low mortality rates (referent category).

Abbreviations: CI, confidence intervals; OR, odds ratio.

© 2023 Bevel MS et al. *JAMA Oncology.*

eTable 2. Results and Discussion

After adjusting for age, race, and poverty rate, we observed an almost 30% increased odds of high obesity-related cancer mortality among counties with high food swamp scores (AOR = 1.29; 95% CI = 1.03 – 1.63). Additional adjustment for physician density per county shows a slightly higher odds of high obesity-related cancer mortality (AOR = 1.32; 95% CI = 1.04 – 1.67). However, after adjusting for adult obesity rates, we did not observe a significant association between high or moderate food swamp scores and high obesity-related cancer mortality. Additionally, we did not show a significant association in fully adjusted models between high or moderate food desert scores and high obesity-related cancer mortality.

After fully adjusting for demographic covariates and adult obesity rates, food deserts and swamps were not significantly associated with obesity-related cancer mortality. The reason is that obesity might be on the causal pathway between our food environment measures and obesity-related cancer mortality, thus being a mediator. According to Rothman et al.[1], potential confounders have to meet three criteria: 1) it must be related to the exposure variable, 2) it must be a risk factor for the disease or outcome variable, and 3) it must not be affected by the exposure or outcome variable. After considering obesity as a potential confounder, it does not satisfy the third criterion because residing in poor food environments affects adult obesity rates. Future analyses should analyze the mediating (direct and indirect) impact of obesity on poor food environments and obesity-related cancer outcomes.

© 2023 Bevel MS et al. *JAMA Oncology.*

© 2023 Bevel MS et al. *JAMA Oncology*.

eTable 3. Fully Adjusted Multilevel Generalized Mixed Effects Models for the Association of Food Environment Measures and Obesity-Related Cancer Mortality among U.S. Counties (N = 3038)

	Adjusted OR (and 95% CI) of Obesity-Related Cancer Mortality					
	Food Deserts			Food Swamps (Comprehensive RFEI)		
**Models**	Low	Moderate	High	Low	Moderate	High
**Model 1**						
Moderate vs. Low	1.00 (Referent)	**1.47 (1.19 – 1.82)**	0.87 (0.70 – 1.09)	1.00 (Referent)	**1.26 (1.02 – 1.57)**	1.26 (1.00 – 1.60)
High vs. Low	1.00 (Referent)	**1.40 (1.11 – 1.76)**	1.05 (0.83 – 1.32)	1.00 (Referent)	1.16 (0.92 – 1.46)	**1.45 (1.14 – 1.85)**
**Model 2**						
Moderate vs. Low	1.00 (Referent)	**1.50 (1.21 – 1.87)**	0.91 (0.72 – 1.15)	1.00 (Referent)	1.23 (0.99 – 1.54)	1.25 (0.98 – 2.60)
High vs. Low	1.00 (Referent)	**1.44 (1.14 – 1.83)**	1.05 (0.83 – 1.33)	1.00 (Referent)	1.12 (0.88 – 1.43)	**1.45 (1.13 – 1.86)**
**Model 3**						
Moderate vs. Low	1.00 (Referent)	**1.46 (1.18 – 1.81)**	0.88 (0.70 – 1.11)	1.00 (Referent)	1.15 (0.92 – 1.43)	1.02 (0.80 – 1.31)
High vs. Low	1.00 (Referent)	**1.38 (1.09 – 1.74)**	1.09 (0.86 – 1.38)	1.00 (Referent)	1.00 (0.79 – 1.27)	1.05 (0.81 – 1.35)
**Model 4**						
Moderate vs. Low	1.00 (Referent)	**1.48 (1.19 – 1.84)**	0.92 (0.73 – 1.16)	1.00 (Referent)	1.11 (0.88 – 1.39)	1.01 (0.79 – 1.30)
High vs. Low	1.00 (Referent)	**1.40 (1.10 – 1.78)**	1.08 (0.85 – 1.38)	1.00 (Referent)	0.95 (0.74 – 1.22)	1.03 (0.79 – 1.34)

Model 1: Adjusted for the percentage of county population ages 65 years old or older, percentage of NH-Black residents per county, and poverty rate per county. Model 2: Adjusted for the percentage of county population ages 65 years old or older, percentage of NH-Black residents per county, poverty rate per county, and physician density. Model 3: additionally adjusted for adult obesity rate per county. Model 4: full model.

Results from this generalized mixed effects models can be interpreted as the log odds of counties with either high or moderate obesity-related cancer mortality rates compared to the log odds of counties with low mortality rates (referent category).

Low categorized as counties with obesity-related cancer mortality rates from 31.0 – 74.0 per 100,000.
Moderate categorized as counties with obesity-related cancer mortality rates from 75.0 – 82.0 per 100,000.
High categorized as counties with obesity-related cancer mortality rates from 83.0 – 185.7 per 100,000.
Abbreviations: CI, confidence intervals; OR, odds ratio.

© 2023 Bevel MS et al. *JAMA Oncology.*

eTable 3. Results and Discussion

After adjusting for age, race, poverty rate, and physician density, we observed a 45% increased odds of high obesity-related cancer mortality among counties with high food swamp scores (AOR = 1.45; 95% CI = 1.13 – 1.86). However, in our fully adjusted models, we did not observe a significant association between high or moderate food swamp scores and high or moderate obesity-related cancer mortality. Conversely, the fully adjusted odds of counties having moderate (AOR = 1.48; 95% CI = 1.19 – 1.84) or high (AOR = 1.40; 95% CI = 1.10 – 1.78) obesity-related cancer mortality was higher among counties with moderate food desert scores. Yet, we did not find a significant association between high food desert scores and moderate or high obesity-related cancer mortality.

The lack of association in the relationship between high vs. low food desert scores and obesity-related cancer mortality could be due to the proportion of counties/county equivalents in the highest level of food desert scores having a higher proportion of persons that are extremely poor and living very far from a grocery store, thus they could be growing and consuming their own produce and livestock. Recall that the definition of food deserts used in the main paper was low income (household income ≤ 200% of the federal poverty threshold) and low access (being more than 1 mile from a supermarket/grocery store in an urban area, or more than 10 miles in a rural area)[2,3]. Typically food deserts are defined as low access to grocery stores, and still it is not specific to the typical lived experiences of citizens compared to our comprehensive food swamp definition (accounting for the ratio of unhealthy food options to healthy food options per county).

© 2023 Bevel MS et al. *JAMA Oncology.*

eReferences.

1. Rothman KJ, Greenland S, Lash TL. *Modern Epidemiology*. Wolters Kluwer Health/Lippincott Williams & Wilkins; 2008.
2. Walker RE, Keane CR, Burke JG. Disparities and access to healthy food in the United States: A review of food deserts literature. *Health Place*. Sep 2010;16(5):876-84. doi:10.1016/j.healthplace.2010.04.013
3. ver Ploeg M, Breneman V, Farrigan T, et al. *Access to Affordable and Nutritious Food: Measuring and Understanding Food Deserts and Their Consequences: Report to Congress*. 2009. *Administrative Publication Number 036*. 2009-06. https://ageconsearch.umn.edu/record/292130/files/12716_ap036_1_.pdf

© 2023 Bevel MS et al. *JAMA Oncology.*
