# Supplementary material for: Association of Food Deserts and Food Swamps With Obesity-Related Cancer Mortality in the US
Source: JAMA Oncol. 2023 May 4;9(7):909–16. doi: 10.1001/jamaoncol.2023.0634 (PMC10160992; doi:10.1001/jamaoncol.2023.0634)
Supplement: Supplement 2. — Data Sharing Statement [file jamaoncol-e230634-s002.pdf]

Data Sharing Statement

Bevel. Association of Food Deserts and Food Swamps With Obesity-Related Cancer Mortality in the US. *JAMA Oncol*. Published May 04, 2023. doi:10.1001/jamaoncol.2023.0634

Data
Data available: No

Additional Information
Explanation for why data not available: The data comes from publicly available sources (CDC and the USDA).
